# The effect of 4-hexylresocinol administration on SCC-9 cells: mass spectrometric identification of proteins and cDNA microarray analysis

**DOI:** 10.1186/s40902-021-00314-6

**Published:** 2021-08-01

**Authors:** Yei-Jin Kang, Seong-Gon Kim

**Affiliations:** Department of Oral and Maxillofacial Surgery, College of Dentistry, Gangneung-Wonju National University, Jibyun-dong, Gangneung, Gangwondo 28644 Republic of Korea

**Keywords:** 4-hexylresorcinol, Oral squamous cell carcinoma, Keratin, Histone

## Abstract

**Background:**

In stress situations, bacteria produce dormancy-inducing factors to stop cell growth. The dormancy-inducing factors may have an inhibitory effect on tumor cell growth. Here we analyzed the differentially expressed protein profiles after 4-hexylresorcinol (4HR), one of the dormancy-inducing factors, administration using in vitro oral squamous carcinoma cells (SCC-9).

**Method:**

The control group was SCC-9 cells culture without 4HR administration. The experimental group received 10 μg/mL of 4HR. Collected proteins from each group were loaded for 2D electrophoresis. Among the separated proteins, 20 differentially expressed proteins were selected and processed for LC-MS/MS.

**Results:**

In proteomic analysis, the expression of keratin 1, keratin 10, and histone H2B were increased. In cDNA microarray assay, the genes related to the cellular differentiation (involucrin, keratin 13, 14) were highly expressed in the 4HR treated group (fold ratio > 2.0; Table 2). Interestingly, histone family was upregulated in the cDNA microarray assay.

**Conclusion:**

The administration of 4HR on SCC-9 cells increased epithelial cell differentiation markers and histone.

## Background

Alkylresorcinols, natural non-isoprenoid lipids found in various plant and bacterial species, attract attention because of a variety of biological functions including non-specific antioxidants, antimutagens, and regulatory molecules of proliferation [1]. Chemical analogs of such lipids exert the anticancer effects as already have been proved in animal models for colon [2], lung [3], and pancreas tumors [4], mononuclear cell leukemia, hepatocellular neoplasms, and circulatory system tumors [5] without clear mechanism. The demonstrations of a potential anti-tumor effect of alkylresorcinols and their non-specificity to various tumors made us eager to undertake studies for elucidating 4-hexylresorcinol (4HR) action on oral squamous cell carcinoma (OSCC).

OSCC is a common malignant cancer, and the overall cure rate has not been improved for decades despite recent development of cancer therapeutics [6]. The drug resistance of OSCC is explained by heterogenous population of OSCC, and it is related to “field cancerization” theory [7]. If the drug has dual functions such as inducing apoptosis of active proliferating cancer cells and redifferentiation of cancer cells which escape from the apoptosis, the therapeutic effects will be increased in the heterogenous OSCC. Therefore, the chemical revertant which can inhibit cancer through induction of cancer differentiation must be developed for OSCC to improve the overall cure rate.

We believe that eukaryotic cells might also produce similar chemical analogs, and these chemicals might help to survive in the micro-environmental stress situation. Starving condition increases the sensitivity to cancer treatment and the survival of normal cells [8]. Therefore, it is reasonable to suggest that mechanisms of 4HR action, same analogy to the regulation of physiological state and activity in microorganisms, will be similar to cancer cells showing inhibition of cell proliferation. Since the anti-tumorigenic effect of 4HR has been suggested by the national toxicology program [9], 4HR-mediated anti-tumor mechanism has been illuminated. 4HR inhibits transglutaminase-2 activity [10] and subsequent nuclear factor-kappaB (NF-κB) signaling pathway [11]. 4HR stimulates the differentiation of oral cancer cells via E2F and Sp1-mediated pathways [12]. 4HR suppresses calcium oscillation in oral cancer cells [13].

The objective of this study was to determine differentially expressed proteins in SCC-9 cells after 4HR administration.

## Methods

### Cell cultures

SCC-9 cells from the American Type Culture Collection (ATCC; Manassas, VA) were grown to confluence in Ham’s F12/Dulbecco’s modified Eagle’s medium (Gibco, BRL, Gaithersburg, MD) containing 1% penicillin/streptomycin, fibroblast growth factor-2 (100 μg/ml), and 10% fetal calf serum (FCS). 4HR (Sigma, St. Louis, MO) was added to confluent cells to final concentrations at 10 μg/mL (50 μM).

### Sample analysis by liquid chromatograph-tandem mass spectrometer (LC-MS/MS)

Analytical 2-D electrophoresis and in-gel proteolytic digestion were followed by Doucette and Li [14] and Gharahdaghi’s method [15], respectively. The final tryptic digested peptides were resolved with 7 μl deionized water (DW) in 0.1% formic acid. DW and HPLC-grade acetonitrile were used for the preparation of eluents. Chromatographic separations were performed using a Nano LC 1D system (Eksigent Technologies, CA, USA). Samples (6 μL) were injected directly onto a 150 um × 150 mm column (Vydac 218MS5, 1515; Grace Vydac, Hesperia, CA, USA) and eluted with a linear gradient of 1–80% acetonitrile (0.1% formic acid) in 120 min. Fused-silica 20 μm i.d. tubings were used for pre- and post-column liquid connections. The spots from each individual digest were analyzed by tandem mass spectrometry (MS/MS) using the QqTOF mass spectrometer (QSTAR XL, Applied Biosystems/MDS Sciex, Foster Citys, CA, USA). The built-in IDA method was used for automatic “Rolling Collision Energy” [16].

### cDNA microarray

cDNA microarray analysis was performed by Genomic Tree Co. (Daejeon, Korea) using Agilent’s human whole genome 4 X 44 K chips (Santa Clara, CA). After12-hour treatment with or without 4HR (10 μg/mL) in SCC-9, total RNA was extracted using TRI Reagent as recommended by the manufacturer (Molecular Research Center, Inc. Cincinnati, OH).

### MTT assay

The respiratory activity in the control and 4HR-treated cultures was assessed as previously described [17]: they were incubated in 6-well multiplates with yellow tetrazolium salt 3-(4, 5-dimethylthiazole-2-yl)-2,5-diphenyltetrazolium bromide (MTT) solution (Cell proliferation kit I; Roche Molecular Biochemicals) for 4 hours at room temperature. Formazan crystals were solubilized overnight, and the product was quantified spectrophotometrically by measuring absorbance at 590 nm using a Victor Multilabel counter (Perkin-Elmer-Wallac, Freiburg, Germany).

## Results

**Fig. 1:**
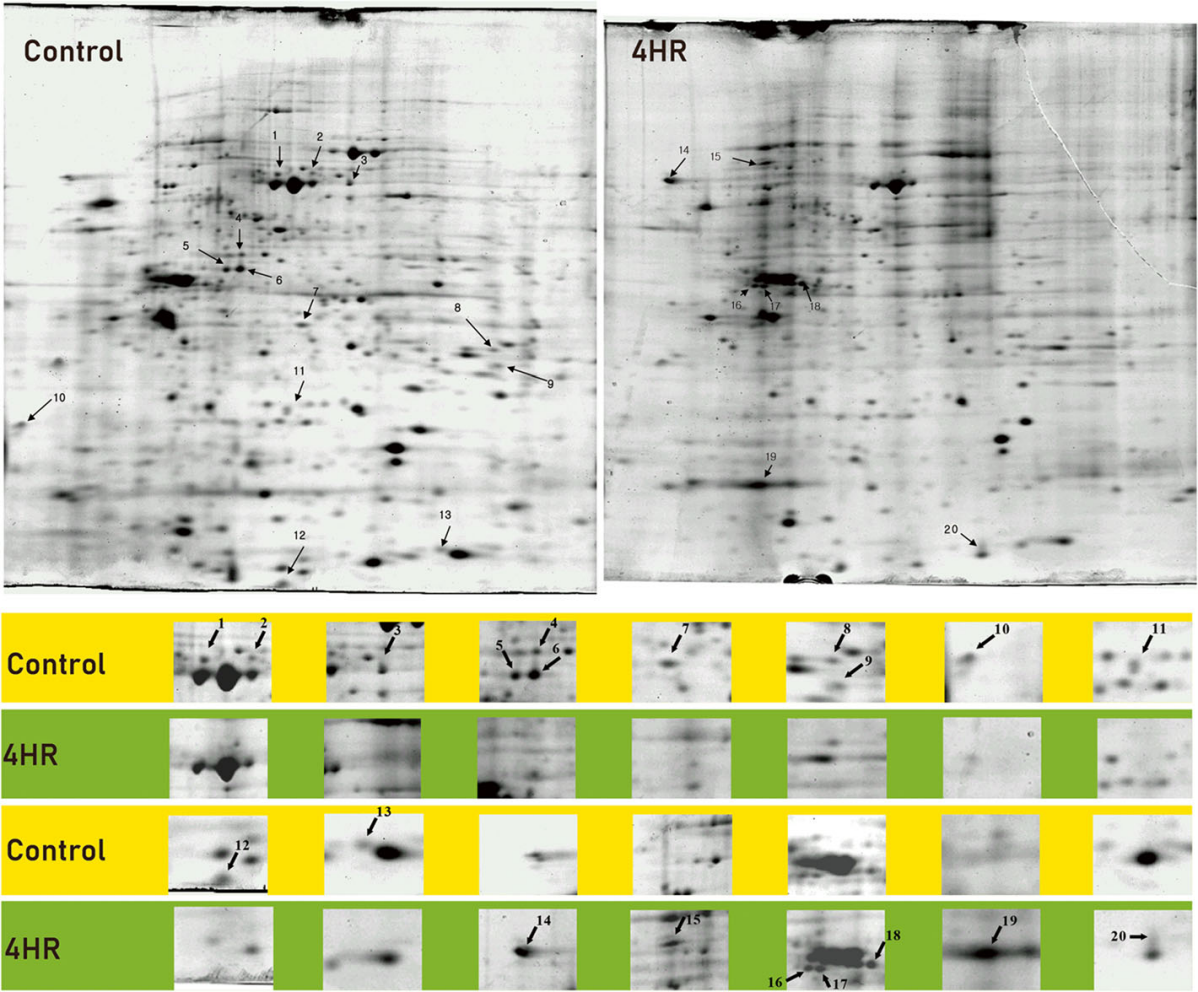
Two-dimensional gel electrophoresis and proteomic analysis. SCC-9 cells were cultured to confluency and treated with 4HR-treated (100 μg/ml) for 24 h. Cell lysates were analyzed by 2D gel electrophoresis. Differential expression protein spots were observed in 2D electrophoregrams from control (**A**, **C**, **E**, **G**) and SCC-9 cells (**B**, **D**, **F**, **H**). Highly expressed keratin 1 (**D**), keratin 10 (**F**), and histone H2B protein (H2BFN) (**H**) in 4HR treated cells were identified by quadrupole-time of flight mass spectrometry. Arrows 1, 2, and 3 indicated keratin 1, keratin 10, and H2BFN, respectively

**Fig. 2:**
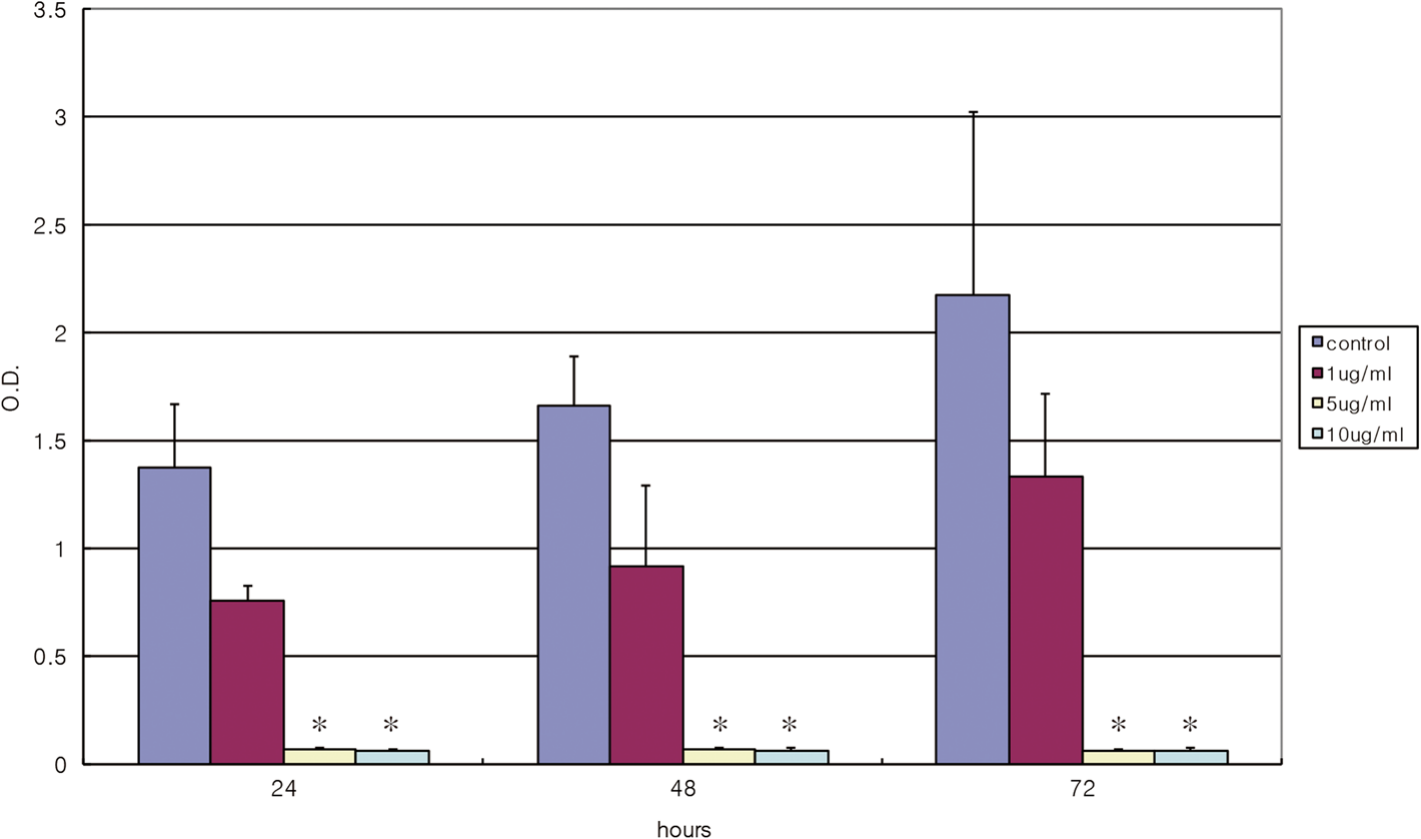
The results of the MTT assay. The cell proliferation assay and the inhibition of respiratory activity. The results of the MTT assay showed a significant decrease in viable cell number in SCC-9 (*P < 0.05 compared to control)

In proteomic analysis, the expression of keratin 1, keratin 10, and histone H2B were increased in 2-D gel electrophoresis and they were identified by Quadrupole-time of flight (Q-TOF) (Fig. 1 and Table 1). Interestingly, keratin 1 showed as 2 spots and they were shown conflicting expression patterns. Spot 8 and spot 14 were identified as keratin 1 (Fig. 1). According to MS analysis, spot 8 showed 8 amino acid matching, and spot 14 showed 12 amino acid matching. The molecular weight of spot 8 was smaller than spot 14. Therefore, highly expressed keratin 1 in the control might be a defective protein. Considering that SCC-9 has poorly differentiated cells, keratin expression could have a defect. In the cDNA microarray assay, the genes related to the cellular differentiation (involucrin, keratin 13, 14) were highly expressed in the 4HR treated group (fold ratio > 2.0; Table 2). Interestingly, histone family was upregulated in the cDNA microarray assay. The genes related to calcium channel and caspase were significantly increased (fold ratio > 2.0). However, the genes related to cell cycle, cellular proliferation, and gene transcription were generally decreased their expression (fold ratio<-2.0). We tested the effect of 4HR on cancer cell proliferation. As shown in Fig. 2, 4HR significantly inhibited cell proliferation with the profound effect from 5 μg/ml (*p* < 0.05).

**Table 1:** The results of two-dimensional electrophoresis and Q-tof

Spot N.	Control intensity	4HB intensity	Gene name	Protein name
1	64,786	0	HNRPK	Heterogeneous nuclear ribonucleoprotein K isoform a
2	44,441	10	HNRPK	Heterogeneous nuclear ribonucleoprotein K isoform a
3	64,032	10	KRT9	Keratin 9
4	49,739	10	HNRPC	Heterogeneous nuclear ribonucleoprotein C isoform b
5	94,978	10	HNRPC	HNRPC protein
6	274,978	10	HNRPC	HNRPC protein
7	34,603	10	KRT9	Cytokeratin 9
8	42,731	10	KRT1	Keratin 1
9	53,724	10	TPI1	Triosephosphate isomerase 1
10	63,649	13,094	PTMS	Parathymosin
11	65,855	10	PRDX2	Peroxiredoxin 2 isoform b
12	93,949	10	Ubc	Ubc protein
13	39,829	7239	S100	Homo sapiens S100 calcium-binding protein A11
14	63,786	304,971	KRT1	Keratin 1
15	2031	116,114	KRT1	Keratin, type II cytoskeletal 1
16	11,423	92,533	NPM1	B23 nucleophosmin
17	10,371	132,731	NPM1	B24 nucleophosmin
18	9385	115,967	NPM1	Nucleophosmin 1
19	3503	584,841	H2BFN	Histone H2B (H2B)
20	100	42,623	KRT10	Keratin 10

**Table 2:** The results of cDNA microarray. The genes of interest were shown among the genes which showed significant changes at 12 h after 4HR application (10 μg/mL)

Title	GenBank	Fold-ratio
**Apoptosis-associated tyrosine kinase**	**AK131529**	**3.395**
**Block of proliferation 1**	**NM_015201**	**2.254**
**Calcium channel, voltage-dependent, T type, alpha 1G subunit**	**NM_018896**	**4.046**
**Caspase 2**	**NM_032982**	**2.697**
**Caspase 6**	**NM_001226**	**2.188**
**Caspase 8**	**NM_033356**	**2.049**
**Collagen type I, alpha 2**	**NM_000089**	**2.168**
**Collagen type IV, alpha 1**	**NM_001845**	**2.334**
**Collagen type V, alpha 2**	**NM_000393**	**2.396**
**H2A histone family, member V**	**NM_012412**	**2.117**
**H2A histone family, member Y**	**NM_138610**	**2.021**
**H3 histone, family 3B (H3.3B)**	**NM_005324**	**2.261**
**Interleukin 1, beta**	**NM_000576**	**2.568**
**Involucrin**	**NM_005547**	**3.176**
**Keratin 13**	**NM_002274**	**2.718**
**Keratin 14**	**NM_000526**	**2.823**
**Cyclin C**	**NM_005190**	**− 3.460**
**Mitogen-activated protein kinase 1**	**NM_002745**	**− 3.663**
**Mitogen-activated protein kinase 13**	**NM_002754**	**− 2.024**
**Mitogen-activated protein kinase 6**	**NM_002748**	**− 3.003**
**Mitogen-activated protein kinase kinase 4**	**NM_003010**	**− 3.497**
**Polymerase (RNA) I polypeptide B, 128 kDa**	**NM_019014**	**− 2.079**
**Polymerase (RNA) II (DNA directed) polypeptide B, 140 kDa**	**NM_000938**	**− 2.421**
**Polymerase (RNA) II (DNA directed) polypeptide K, 7.0 kDa**	**NM_005034**	**− 3.165**
**Polymerase (RNA) III (DNA directed) polypeptide B**	**NM_018082**	**− 2.041**
**Serine threonine kinase 39**	**NM_013233**	**− 2.075**
**Serine threonine kinase 17a**	**NM_004760**	**− 2.016**
**Serine threonine kinase 38**	**NM_007271**	**− 3.413**
**Transforming growth factor, alpha**	**NM_003236**	**− 2.513**

## Discussion

In this study, 4HR accelerated carcinoma epithelial cell differentiation showing upregulation of involucrin and keratins expression (Fig. 1 and Table 2). 4HR increased the expression of histone family in both proteomic analysis (Fig. 1) and cDNA microarray assay (Table 2). Although this study focused on SCC-9 cells and OSCC, we also observed the inhibitory action of 4HR on breast carcinoma cell lines [18]. Besides, 4HR inhibited cell proliferation-related genes including cyclin C and other RNA polymerases by cDNA microarray assay (Table 2). Cyclins may be an important target for 4HR mediated antiproliferative actions. Supportively, 4HR also inhibits cell proliferation of *ras* oncogene transformed fibroblasts not in normal fibroblasts [19].

4HR also increases epithelial cell differentiation in SCC-9 cells showing the upregulation of various keratins and involucrin, which is observed both in vitro SCC-9 cell culture system and *in vivo* SCC-9 cell implanted xenograft model [12]. We further confirmed the upregulation of keratins and involucrin by cDNA microarray and proteomic analysis (Fig. 1 and Table 2). Interestingly, 4HR increased several voltage-dependent calcium channels (Table 2). Indeed, 4HR stimulates intracellular calcium uptake [13]. 4HR mediated increase of calcium uptake is due partly to the upregulation of calcium channels (Table 2). Finding the evidence of tumor cell differentiation is very important because it will minimize host damage induced by conventional therapy. Therefore, the study on the chemically induced tumor differentiation should be encouraged. However, it is far behind to explain the mechanism of 4HR mediated acceleration of SCC-9 cell differentiation.

Mechanistically, 4HR mediated various effects on cancer cells may be due in part to the different expression of unidentified 4HR receptors in tumor cells compared to control cells. We do not know the 4HR receptor and its signal transduction pathways except the increase of calcium level at this moment [13]. The increase of calcium uptake by 4HR is the only known phenomenon at this moment with regard to the signaling pathway [13]. Intracellular calcium is broadly related to cell proliferation, differentiation, and apoptosis. Blocking of calcium channels attenuated 4HR mediated antiproliferative and apoptotic effects on cancer cell lines [13]. Interestingly, the elevated calcium level by 4HR seems similar to the increase of intracellular content of calcium in bacterial cells by 4HR [20]. An increase of calcium uptake by 4HR may be an essential step for various biological effects on cells. Importantly, an increase in the intracellular calcium may result in changes of the expression of differentiation markers [21]. According to the known concepts, tumorigenesis is a cellular dedifferentiation and the intracellular signal pathway is directed to the uncontrolled growth [22]. Tumor growth and development are coupled with down-regulation of differentiation-related calcium binding or modulating genes [23], so it can be assumed a positive relationship between tumorigenesis and down-regulation of these genes. Regarding that, the cellular differentiation is largely dependent on calcium [24] and the observed effects of 4HR is due to the increase of its intracellular level in SCC-9 cells. Revertant function of 4HR may be dependent on the increase of calcium uptake because an increased calcium uptake stimulates the cellular differentiation in the normal keratinocytes [24, 25]. Other signal molecules or downstream target molecules may play an important role for 4HR-mediated cellular effects. Protein kinase C-α, for example, plays an important role in calcium-induced keratinocytes differentiation [26]. It is definitely necessary to pursue the precise molecular mechanism of 4HR mediated antitumor effects.

Noteworthy, the action of 4HR is dose-dependent: being introduced at relatively high doses (5-10 μg/mL), 4HR caused the rupture of cellular membranes in cells and possessed cytotoxic action [12]. This is similar to the effect of conventional anti-cancer drugs that are usually toxic and induce apoptosis in cancer cells where DNA looks as fragmented material and protein synthesis is inhibited [27]. However, the antiproliferative effect of 4HR at low concentration (1 μg/mL) is not followed by appearance of cytotoxicity or apoptosis signs [12]. In similar concentrations (0.1-1 mM), 4HR and resveratrol are not cytotoxic to human lymphocytes [28]. As for the potential use of 4HR as an anti-cancer drug, we have to mention the previous toxicology and carcinogenesis studies demonstrating that the oral administration of 4HR in doses up to 650–1000 mg/kg to animals unaffected their survival [9]. In the Zebra fish study, the administration of 4HR in doses up to 1 mM (194 μg/ml) is not teratogenic to the developing embryo [18]. The effective dose of 4HR, used for injections of carcinoma-grafted nude mice, was 10 mg/kg body weight, i.e., two orders of magnitude less, and caused no toxic side effects in our in vivo experiments [12]. They showed the similar action of 4HR on SCC-9 cells xenografted nude mice model, that is, the significant deceleration of tumor growth and the elicitation promotion of cellular differentiation followed by the formation of keratinous matrix in which individual cells underwent contact inhibition [12].

The clinical use of 4HR or cognate substances should require a careful selection of active doses. This is important not only to controlling the type of biological effect but also to avoid a side effect since a prolonged use of 4HR may cause nephropathy and osteosclerosis [29], which can be explained based on 4HR-induced increase of intracellular calcium [13]. Historically, 4HR was used for the therapeutic drug for infectious disease and its solution in olive oil had been taken by human without any complication [27, 28]. Now, it is a component of topical antiseptics on oral mucosa (C0691518: information was derived from the NIH UMLS). In some bacterial species, extracellular alkylresorcinols play as regulatory factors, are accumulated in developing microbial cultures and, when reaching a threshold level, entering them to the stationary phase. Further increase in alkylresorcinol concentration elicits entering of bacteria to a dormant state followed by the formation of morphologically distinct cyst-like cells [30]. Similar analogy, opportunistic pathogens, such as *Pseudomonas aeruginosa*, produce N-(3-oxo-dodecanoyl) homoserine lactone which selectively impairs the regulation of NF-kB functions in activated mammalian cells [31].

## Conclusion

In this study, the administration of 4HR on SCC-9 cells increased epithelial cell differentiation markers such as involucrin and keratins. The expression of histone was also increased by 4HR administration.

## Data Availability

Data sharing is not applicable to this article since no dataset was generated or analyzed during the current study.

## Abbreviations

4HR: 4-hexylresorcinol
OSCC: Oral squamous cell carcinoma
NF-κB: Nuclear factor-kappaB
LC-MS/MS: Liquid chromatograph-tandem mass spectrometer
